# Exposure assessment during paint spraying and drying using PTR-ToF-MS

**DOI:** 10.3389/fpubh.2023.1327187

**Published:** 2024-01-12

**Authors:** Srdjan Sabic, David Bell, Bojan Gasic, Kaspar Schmid, Thomas Peter, Claudia Marcolli

**Affiliations:** ^1^Institute for Atmospheric and Climate Science, ETH Zurich, Zurich, Switzerland; ^2^Laboratory of Atmospheric Chemistry, Paul Scherrer Institute, Villigen, Switzerland; ^3^Swiss State Secretariat for Economic Affairs (SECO), Chemicals and Occupational Health, Bern, Switzerland

**Keywords:** spraying applications, workplace exposure, proton transfer reaction time-of-flight mass spectrometry, volatile organic compounds, exposure models

## Abstract

Spraying is a common way to distribute occupational products, but it puts worker's health at risk by exposing them to potentially harmful particles and gases. The objective of this study is to use time-resolved measurements to gain an understanding of spray applications at the process level and to compare them to predictions of exposure models. We used proton transfer reaction time-of-flight mass spectrometry (PTR-ToF-MS) at 1-s time resolution to monitor the gas phase concentration of the solvents acetone, ethanol, butyl acetate, xylene and 1-methoxy-2-propy acetate during outdoor spraying and indoor drying of metal plate under various conditions of outdoor air supply. We found that during spraying, gas-phase exposure was dominated by the more volatile solvents acetone and ethanol, which exhibited strong concentration variations due to the outdoor winds. During drying, exposure strongly depended on the strength of ventilation. Under conditions with high supply of outdoor air, our measurements show a near-exponential decay of the solvent concentrations during drying. Conversely, under conditions without outdoor air supply, the drying process required hours, during which the less volatile solvents passed through a concentration maximum in the gas phase, so that the exposure during drying exceeded the exposure during spraying. The concentrations measured during spraying were then compared for each of the substances individually with the predictions of the exposure models ECETOC TRA, Stoffenmanager, and ART using TREXMO. For these conditions, ECETOC TRA and Stoffenmanager predicted exposures in the measured concentration range, albeit not conservative for all solvents and each application. In contrast, ART largely overestimated the exposure for the more volatile solvents acetone and ethanol and slightly underestimated exposure to 1M2PA for one spraying. ECETOC TRA and ART do not have options to predict exposure during drying. Stoffenmanager has the option to predict drying together with spraying, but not to predict the drying phase independently. Our study demonstrates the importance of considering both the spray cloud and solvent evaporation during the drying process. To improve workplace safety, there is a critical need for enhanced exposure models and comprehensive datasets for calibration and validation covering a broader range of exposure situations.

## 1 Introduction

Spraying is a widespread application to disperse consumer and occupational products uniformly in air or on surfaces. Typical occupational uses include spraying of lacquers or paints, pesticides, wood preservatives, detergents, or disinfectants (1). Health hazards may arise from dermal exposure or inhalation of particles and gases during spraying. To ensure uniform distribution by spraying, the products are dissolved or suspended in a solvent or a solvent mixture. During application, the solvents evaporate from the sprayed surfaces, resulting in additional exposure to the vapors if workers remain in the area during the drying phase. Therefore, in spray applications, the primary exposure to the spray cloud is followed by a secondary exposure to the vapors emitted by droplets or by treated surfaces. Solvent evaporation from surfaces is also part of many wiping, brushing, rolling, or mopping applications as required in painting, lacquering, polishing, or cleaning of surfaces.

The level of exposure reached during drying of sprayed surfaces depends on factors related to the product's composition and on workplace conditions. Product-related properties are the vapor pressure of the solvents, their concentration in the product, and their miscibility with the other mixture components. The most relevant workplace properties are room size, ventilation or air exchange rate, position of the workers with respect to the emission source, and the protection measures taken, for instance with respect to duration of the occupational task.

Under the European Chemicals Act Registration, Evaluation, Authorization and restriction of Chemicals (REACH), companies are obliged to register all substances they intend to sell on the European market (2–4). Since the inception of REACH in 2007, the European Chemical Agency (ECHA) has provided safety data for a wide array of individual substances, most of which are freely accessible. In Switzerland, the safety data sheets provided to the customers together with the products include maximum allowable concentrations (MAK—“Maximale Arbeitsplatzkonzentration”) for short term (15 min) and day shift (8 h) exposures (see www.suva.ch). Another parameter is Derived No-Effect Level (DNEL) that constitutes an essential toxicological exposure threshold necessitated for the assessment of chemicals seeking market entry within both the Swiss and EU regulatory frameworks, and both parameters (MAK and DNEL) are covered under the umbrella term Occupation Exposure Limits (“OEL”).

To estimate whether workplace exposures exceed DNEL values, ECHA recommends the use of exposure models in a tiered approach (3, 5). Tier 1 models should provide a conservative exposure estimate requiring only a few input parameters. The most widely used Tier 1 model in Europe is European Centre for Ecotoxicology and Toxicology of Chemicals Targer Risk Assessment (ECETOC TRA) (6, 7). The higher tier models Stoffenmanager [Tier 1.5; (8, 9)] and Advanced REACH Tool (Tier 2;ART) (10) are recommended when safe use of the substance cannot be demonstrated based on the initial Tier 1 assessment (3). Yet, intercomparison of these models in different exposure situations revealed significantly different exposure estimates, which would entail disparate safety measures (4). Especially Tier 1 models did not always prove to be the most conservative, an outcome that questions the tiered workflow and rather suggests the use of multiple models to avoid exposure scenarios where safety measures are not sufficient to adequately control the risks. Therefore, to facilitate and unify the simultaneous use of different exposure models, the Translation of Exposure Models (TREXMO) tool has been developed, which includes among others ECETOC TRA, Stoffenmanager, and ART (11–13).

The different exposure models have been summarized and compared in different validation studies [e.g. (4, 14–17), which have revealed systematic under- or overprediction of exposure levels for specific models depending on exposure situations. There is consensus that further validation with more comprehensive datasets covering a broader range of exposure situations is required. Specifically, spraying applications are poorly represented. In a recent review, Hahn et al. (1) identified the need to extend mechanistic model approaches to cover combined exposure to the spray cloud and to solvent evaporation during the drying process. Yet, exposure measurements suited to improve exposure models are scarce.

Input data for model development (e.g., 8) and validation are mostly task- or shift-based exposures at workplaces [e.g., (14, 18, 19). For volatile substances, sorbent-based air sampling is used followed by isolation and identification by gas chromatography coupled with mass spectrometry (GC-MS) (20, 21). This method provides integrated exposure over the entire sampling period. Therefore, no mechanistic understanding of exposure arising from spraying and drying can be derived from such data. Time-resolved measurements are required to gain an understanding at the process level.

A method for online monitoring of volatile organic compounds (VOC) in real-time is proton transfer reaction time-of-flight mass spectrometry (PTR-ToF-MS) (22, 23). This method has become popular in different research fields, e.g., in atmospheric sciences for indoor and outdoor air-quality monitoring and emission studies (24–26), in food and flavor sciences (27, 28), and in medical sciences for real-time breath analysis (29, 30). It has also been successfully applied to workplace exposure for α-diketones in coffee roasteries and breweries (31) and for VOC measurements related to building disinfection during COVID-19 (32).

Under ideal conditions, PTR-ToF-MS uses proton-transfer reaction with H_3_O^+^ for soft ionization to minimize molecule fragmentation, such that the molecular ion at m/z = MW (molecular weight) + 1 can be used as molecular identifier for VOCs. Due to the high mass resolution of the time-of-flight analyzer, peaks of the same mass but with different elemental composition can be discriminated (26). As PTR-ToF-MS enables continuous monitoring of VOCs at a time resolution of 1 Hz, the evolution of mass peaks in mass spectra can be assigned to specific activities. Nevertheless, because mass peaks are not unique for a specific compound, reliable identification of substances requires additional compositional information e.g., from the safety data sheet of the product. Moreover, calibration of each compound is required for quantitative evaluation of the mass spectra when the proton transfer reaction rate is not known.

In this study, we applied PTR-ToF-MS to investigate workplace exposure to a spray paint/lacquer containing five solvents in real-time. We sprayed a black paint onto a metal plate to monitor the spray cloud and the subsequent evaporation from the plate. To simulate near-field conditions, we placed the inlet of the PTR-ToF-MS at a distance to the metal plate that corresponds to the breathing zone of a worker (< 1 m). We monitored the concentration of all five solvents in the spray, namely acetone, ethanol, butyl acetate, xylene, and 1-methoxy-2-propyl acetate (1M2PA) and compared the measured exposures with the values predicted by the exposure models ECETOC TRA (v3), Stoffenmanager (v4.0), and ART (v1.5).

## 2 Materials and method

### 2.1 Spray paint experiments

The paint used for our experiments was “Lackspray schwarz matt RAL 9005” (Albert Berner Deutschland, GmbH). The composition of the paint in terms of weight percentage according to the safety data sheet (SDS) version 07.03.2017/0013 is summarized in Table 1, including the calculated mole fractions. The listed mole fractions exclude the propellants (butane, propane, and dimethyl ether), so that the solvents ethanol, acetone, xylene, butyl acetate (BA), and 1-methoxy-2-propyl acetate add up to the entire composition. Two sets of conversions were done, one considering the lower limit (mole fraction min) and one with the upper limit (mole fraction max) of the composition range to cover the uncertainty in composition.

**Table 1:** Composition of the spray can paint in wt% and its conversion to mole fraction neglecting propellants and substances present only in traces (< 1 %).

**Composition**	**Weight percentage (wt %) (Min)–(Max)**	**Mole fraction min (Min)**	**Mole fraction max (Max)**	**Molecular weight**
Acetone	20–40	0.87	0.68	58.08
Ethanol	1– < 5	0.05	0.11	46.07
Butyl acetate	1– < 10	0.03	0.08	116.16
Xylene	1– < 10	0.02	0.09	106.16
1-Mehoxy-2-propyl acetate (1M2PA)	1– < 5	0.03	0.04	132.16
Butyl glycollate	0.01– < 1	-	-	132.16
Oleic acid, compound with (Z)-N-octadec-9-enylpropane-1,3-diamine (2:1)	0.001– < 0.1	-	-	-
Butane	10–20	-	-	58.12
Propane	5–15	-	-	44.09
Dimethyl ether	10– < 20	-	-	46.07

**Figure 1:**
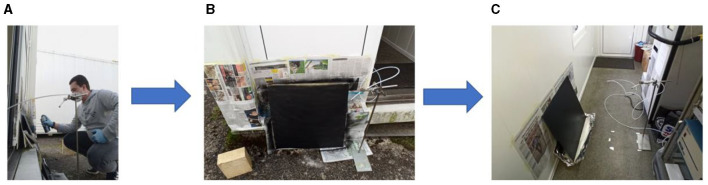
Images illustrating the spraying experiment: **(A)** Outdoor spraying of the product; **(B)** Painted metal plate (outdoors); **(C)** Drying of the paint (indoors).

A metal sheet (64 cm x 64 cm) was sprayed with the spray can for 1–2 min until the surface was evenly covered using the recommended pulse spraying method, which involved dispensing short bursts of paint (see Figure 1A). The weight of the spray can was measured before and after each spraying to derive the amount of sprayed paint. Spraying was conducted outdoors, and the painted metal plate was subsequently moved indoors. During both the spraying and drying process, the PTR-ToF-MS (PTR-ToF-MS-8000, Ionicon Analytik, Austria) inlet was positioned at 30 cm (± 5 cm) from the plate to align with the workplace terminology's definition of a breathing zone [Comité Européen de Normalisation (CEN) (1998) EN1540 Workplace Atmospheres – Terminology] (see Figures 1A, C for illustration). Figure 1B, shows an image of the experimental setup employed for the spraying application. We conducted three independent spraying experiments, each with different strengths of outdoor air supply. The sprayed mass was 90 g for the first, 66 g for the second, and 85 g for the third spraying (as demanded by establishing a uniform layer of paint by spraying under outdoor conditions). The drying took place in a container with a volume of 26 m^3^ (a description of the container is provided in Supplementary material), which was kept at a constant temperature of 25°C using three air conditioning units (model AK 7540, Suter Technik AG, Switzerland). Note that the installed air conditioning just regulated indoor temperature and led to internal ventilation but did not provide exchange with outdoor air. The first drying experiment was with door fully open (90 cm in width and 200 cm in height), resulting in significant exchange with outdoor air. The second drying experiment had a partially open door (with a slit of 4 cm) to limit the exchange of air. Finally, the third drying experiment was with closed door, ensuring negligible exchange with outdoor air. During the drying nobody was inside the container.

### 2.2 Real-Time VOC gas composition measurements with PTR-ToF-MS

We used a high-resolution PTR-ToF-MS to measure gaseous emissions during spraying with the spray paint and during drying of the sprayed surface. The operational details of the instrument have been previously published (22, 23, 33). The ion drift tube was set to standard conditions with a total voltage ranging from 550 to 600 V and a pressure of 2.4 mbar. To maintain a consistent ratio of electric field (*E*) to number density (*N*) of buffer gas molecules in the drift tube (*E*/*N*), we kept values within the range of 119–120 Td during spraying measurements and 111–112 Td during calibration measurements. These variations in E/N were not on purpose, yet the differences are relatively small (6 %) and within the overall uncertainty of the experiment. The Townsend, symbol Td, is a physical unit of *E*/*N*. This ratio is important, because it determines the mean energy of electrons, and hence the degree of ionization. It means that increasing the electric field (units V/m) by some factor has the same consequences as lowering the gas density (units cm^−3^) by the same factor. The Townsend is defined as 1 Td = 10^−17^ V cm^2^. These settings ensure that the ion drift is predominantly influenced by the H_3_O^+^ cluster rather than higher mass water clusters.

The proton transfer reaction can be written as:


(1) $$\begin{array}{l} H_{3}O^{+}\;+\;R\;\to \;RH^{+}\;+\;H_{2}O, \end{array}$$

Here, R denotes the VOC being measured, while RH^+^ represents the protonated molecule detected by the TOF-MS (Equation 1).

### 2.3 Calibration measurements with saturated airflows of the pure solvents

**Figure 2:**
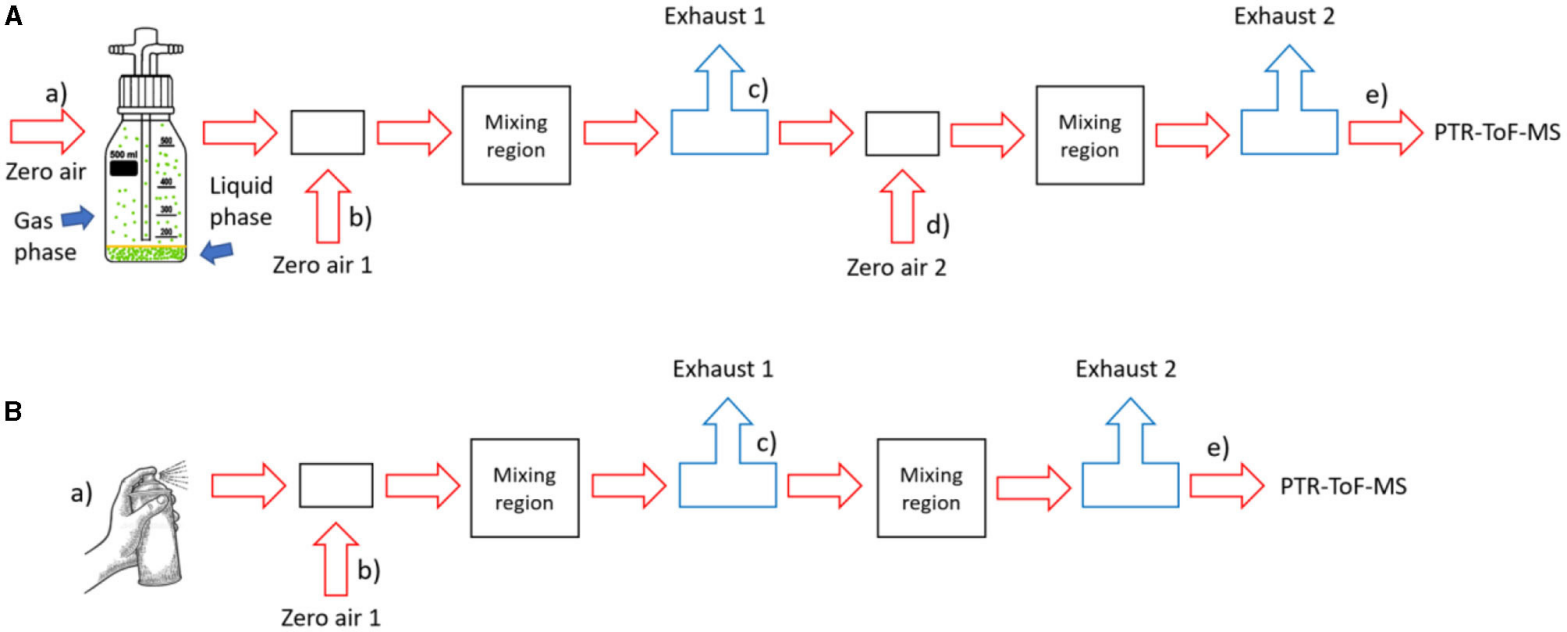
Measurement setup: **(A)** for reference measurements with pure solvents; **(B)** for spraying experiments. More details are given in SM. The lowercase alphabet letters, enclosed in brackets, denote the position of the mass flow controllers, with the sole exception being the notation “a)” within the B section of the illustration: in this position there was no mass flow controller.

To quantify the gas-phase emissions of the spray can paint during spraying and drying, we performed reference measurements with airflows saturated with the five pure solvents obtained from Sigma-Aldrich. We have purchased acetone (ACS reagent with purity of ≥ 99.5 %), ethanol (for molecular biology), xylene (xylenes, isomers plus ethylbenzene, reagent grade), butyl acetate (purity of 99.5%), and 1-methoxy-2 propyl acetate (purity of ≥ 99.5 %). The measurement setup is outlined in Figure 2, setup A. We equilibrated each solvent in a 0.5 L Schott bottle for up to 30 min with closed inlet and outlet lines. Once equilibrium between the gas and the condensed phase was established, air with a flow rate of 0.03–0.05 L/min was passed through the bottle. Due to the high vapor pressures of the pure solvents, we introduced two dilution stages to keep the solvent signals within the linear PTR-ToF-MS measurement range, and two mixing regions (widened part of the metal tubing) to ensure better mixing. Dilution factors (*DF*) were calculated as given in Equation (2).


(2) $$\begin{array}{l} DF_{X}=\frac{f_{X}^{sat}+f_{1}^{zero}}{f_{X}^{sat}}\times \frac{f_{X}^{sat}+f_{1}^{zero}+f_{2}^{zero}-f_{1}^{exh}}{f_{X}^{sat}+f_{1}^{zero}-f_{1}^{exh}}, \end{array}$$

where $f_{X}^{sat}$ is the air flow saturated with species X from the bottle, $f_{1}^{zero}+f_{2}^{zero}$ are the flows of zero air entering the main flow line at positions 1 and 2, respectively, and $f_{1}^{exh}$ is the flow through the exhaust at position 1. Input parameters are presented in Supplementary Table S1 and the resulting *DF*_X_ are listed in Table 2.

**Table 2:** Ion peaks used for evaluation, dilution factors (*DF*) used for calibration and spray paint measurements, calibration factors (*CF*) derived for the selected ion peaks, and saturation vapor pressures (ECHA webpage: https://echa.europa.eu/home).

**Substance**	**Ion peaks m/z**	**Dilution factor (*DF*)**	**Calibration factor (*CF*)**	**Vapor pressures at 25°C [Pa]**
Acetone	60.05	30,401	1.02	32,130
Ethanol	45.03	30,401	5.9	16,926
Butyl acetate	117.05	7,525	1,898	1,200
Xylene	107.08	30,401	4.0	1,048
1M2PA	133.08	602	884	517
Spray can paint	-	168	-	

Additionally, for each solvent, we have measured a lower concentration in a separate setup (injecting a defined amount into a chamber). This process confirmed the obtained calibration factor and helped minimize uncertainties (description of the chamber experiments and the calibration factors derived from them are presented in the Supplementary material). For the spraying experiments, we use the calibration factors from the bottle experiments as they give a lower limit of the concentrations. Moreover, we have corrected for the transmission efficiency (the corresponding curve and equation is presented in the Supplementary Figure S1).

### 2.4 Application to the measurements of complex mixtures

Because of the complexity of the mass spectra of the spray paint with overlapping ion signals from the five solvents, we rely on just one ion peak for each substance in our evaluation. For xylene and 1M2PA we chose the peaks of the parent ions, which are C_8_H${\;}_{11}^{+}$ (m/z = 107.08) and C_6_H_13_O${\;}_{3}^{+}$ (m/z = 133.08), respectively. As the high vapor pressure of acetone leads to a very strong signal of the parent ion peak, which was outside the linear range of the instrument despite dilution, the isotope peak of the parent ion at m/z = 60.05 (^13^C-C_2_H_7_O^+^) was used to ensure linearity of the PTR-MS signal because we observed that the signal of the acetone parent ion at m/z = 59 was above the linearity range of the instrument recommended by the manufacturer as it exceeded 3 ppm even after dilution, which is just above linearity range of the instrument. During spraying, we observed a decrease of the H_3_O^+^ intensity by 5–10 % associated with the peaks that exceeded the linearity range of the instrument even without saturating the detector. As the measured parent ion peak intensity *I*_117.05_ of butyl acetate at m/z = 117.05 also contained shares of a major fragment of 1M2PA, we subtracted the contribution of the 1M2PA fragment, equaling 0.672 of the measured intensity of the parent 1M2PA ion peak (0.672 × *I*_133.08_). This yields a net butyl acetate signal with intensity of *I*_117.05_ – 0.672 × *I*_133.08_.

Ethanol was the most difficult substance to quantify during spraying as its parent ion peak and all its fragments overlap with the propellant dimethyl ether of the spray can paint. We chose the mass peak at m/z = 45.03 (C_2_H_5_O^+^), which proved to be the highest signal in the calibration measurements with pure ethanol and at the same time specific for ethanol in the solvent mixture. However, we needed to exercise caution due to the interference caused by dimethyl ether. This interference could potentially lead to an overestimation of the concentration measured during spraying, owing to the presence of dimethyl ether and fragments from other components in the spray paint. Therefore, we chose to represent m/z = 45.03 as an upper limit for ethanol. The evaluation of the ethanol concentration during drying, on the other hand, should not have been affected by interference from dimethyl ether because we transferred the plate inside the container for spraying while the overspray cloud and propellants remained outside. The resulting dilution factors (*DF*) and the calibration factors (*CF*) obtained by comparing the partial pressures derived from the intensity of the selected peaks with compiled vapor pressures are presented in Table 2.

### 2.5 Exposure assessment with occupational exposure models

We compared our measurements with predictions from Tier 1–2 exposure models available in TREXMO 2.0, specifically ART (version 1.5), Stoffenmanager (version 4.0), and ECETOC TRA (version 3). We used the option to run them all individually within TREXMO (without translation tool), thus avoiding any ambiguity through automatically translating between models. The relevant information for the source term, activity term, and control term are listed in Table 3. Note that in ECETOC TRA the concentrations cannot be inserted exactly but are just selected as >25 %, 5–25 %, 1–5 %, or < 1 %.

**Table 3:** Exposure model parameters set for the exposure assessment of the spraying application.

**Source term (variables)**	**Input for models**	**Model**
State of the substance	Liquid	All models
Vapor pressure	Substance specific (see Table 2)	All models
Concentration present in the product	Substance specific	All models
Mole fraction	Substance specific (see Table 1)	ART
Activity coefficient	Substance specific ^**a**^	ART
Molecular weight	Substance specific (see Table 1)	ECETOC TRA, ART
Distance from the source	Less than 1 m (near-field zone)	All models
Workshop cleaning and maintenance**/**Surface contamination	No daily cleaning of workshop	All models
**Activity term**	**Input for models**	**Model**
Number of employees carrying out the same task simultaneously	1	Stoffenmanager
Task followed by evaporation	Yes (far-field exposure possible)	Stoffenmanager
Type of handling**/**Select process category (PROC)**/**Activity class	Handling of liquids using low pressure low speed and on medium sized surfaces/PROC 11: non-industrial spraying/Spray application of liquids	All models
Task duration	480 min/> 4 h	All models
Type of setting	Professional	ECETOC TRA
Activity sub-class	Surface spraying of liquids	ART
Situation which best represents activity	Moderate application rate (0.3–3 L/min)	ART
Direction of spraying	Only horizontal and downward spraying	ART
Spray technique	Spraying with no or low compressed air use	ART
**Control term**	**Input for models**	**Model**
Select the volume of working room**/**Ventilation**/**Exposure site	Outdoors	All models
Select available control measures**/**localized controls	No control measures at source	ART and Stoffenmanager
Select personal protective equipment	No protection	Stoffenmanager and ECETOC TRA
Distance of exposure source from the building	Close to building	ART

^a^To calculate activity coefficients, we used AIOMFAC, an online tool readily available online (www.aiomfac.caltech.edu).

AIOMFAC was used to determine activity coefficients for exposure assessment with ART. We considered the lower limit (mole fraction min) and upper limit (mole fraction max) of the composition range as input for TREXMO and to calculate activity coefficients with AIOMFAC.

To estimate the combined exposure to the solvent mixture, we calculate the sum index (*SI*) from the individual MAK values using the following formula:


(3) $$\begin{array}{l} SI=\frac{C1}{MAK1}+\frac{C2}{MAK2}+\frac{C3}{MAK3}+\frac{C4}{MAK4}+\frac{C5}{MAK5}, \end{array}$$

where *C1–C5* are the concentrations of the five solvents and *MAK1–MAK5* their MAK values (Equation 3).

## 3 Results

**Figure 3:**
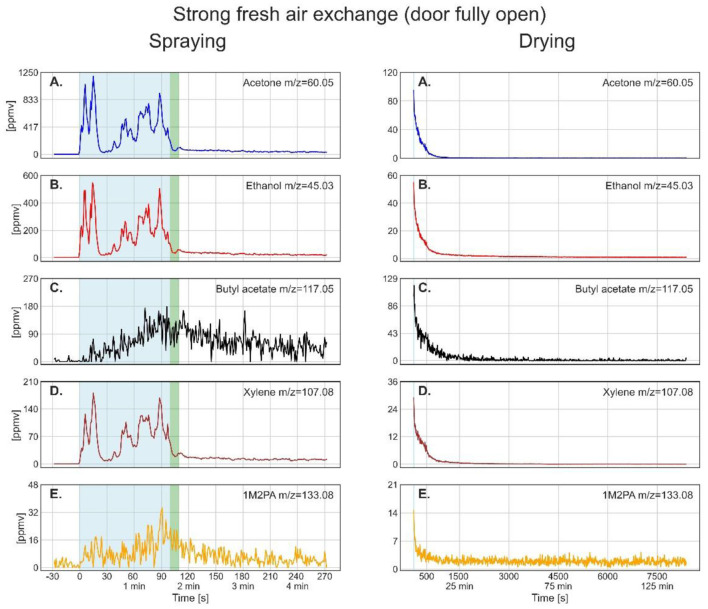
Time series of the monitored solvents in the order of their vapor pressures (from high to low) for the spray paint application with open door during drying. In the left column, measurements during the spraying phase are shown. The drying phase, in the right column, started at 108 s. The light blue shaded regions in the left column mark the effective spraying period. The green shaded area represents the transfer of the metal plate and PTR-ToF-MS inlet from outdoors into the walk-in container. The thin light blue segments in the right column denote the maximum of solvent concentration during drying, for which the average concentrations are given in Table 4.

**Figure 4:**
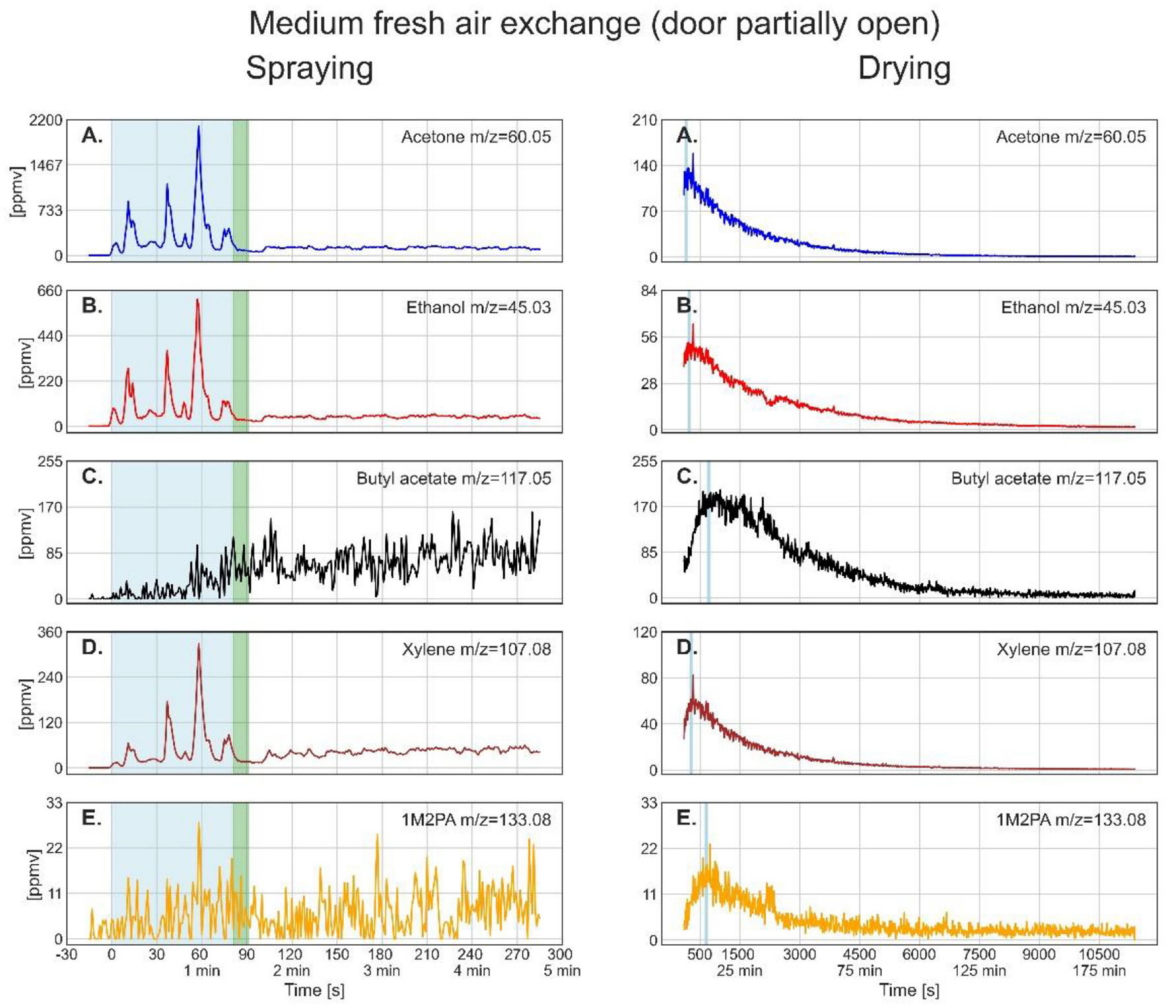
Time series of the monitored solvents in the order of their vapor pressures (from high to low) for the spray paint application with partially open (4 cm) door. In the left column, measurements during the spraying phase are shown. The drying phase, in the right column, started at 98 s. The light blue shaded regions in the left column mark the effective spraying period. The green shaded area represents the transfer of the metal plate and PTR-ToF-MS inlet from outdoors into the walk-in container. The thin light blue segments in the right column denote the maximum of solvent concentration during drying, for which the average concentrations are given in Table 4.

**Figure 5:**
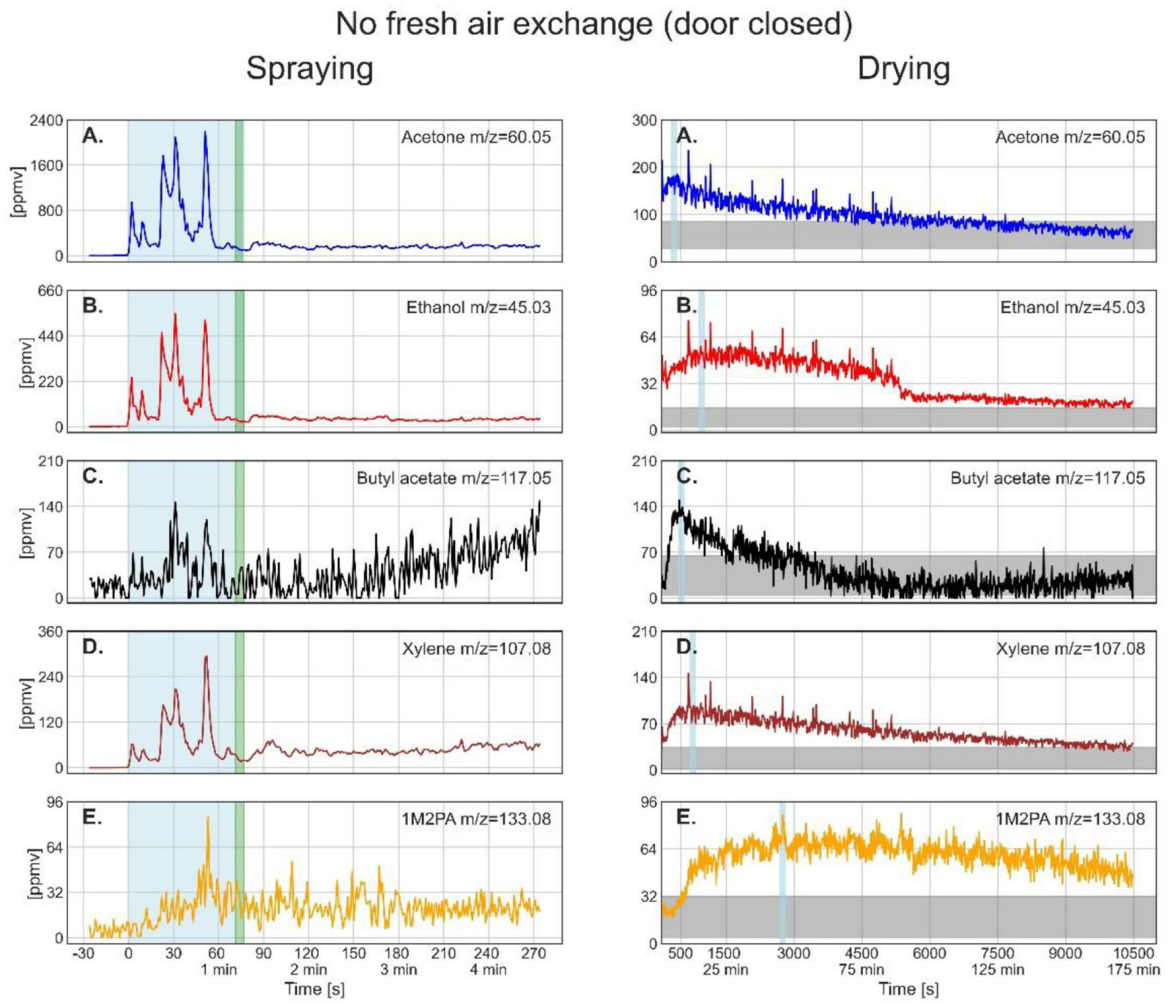
Time series of the monitored solvents in the order of their vapor pressures (from high to low) for the spray paint application with closed door during drying. In the left column, measurements during the spraying phase are shown. The drying phase, in the right column, started at 77s. The light blue shaded regions in the left column mark the effective spraying period. The green shaded area represents the transfer of the metal plate and PTR-ToF-MS inlet from outdoors into the walk-in container. The thin light blue segments in the right column denote the maximum of solvent concentration during drying for which the average concentrations are given in Table 4. The horizontal gray bar shows the solvent concentration calculated for homogeneous distribution within the container after full evaporation from the plate assuming an airtight room and no wall loss.

Figures 3–**5** show the time-resolved concentrations of the five solvents in the spray paint evaluated based on the ion peaks listed in Table 2. The measurements are divided into the spraying phase (left columns) performed outdoors in front of the container, followed by the drying phase (right columns), which took place within the container. Note that the spraying is shown with the instrument time resolution of 1 second, while for the drying, the data was smoothed by taking 10 seconds averages. The green sections after the spraying period mark the transfer of the plate into the container and the re-installation of the inlet in front of the plate at a distance of 30 cm. The drying period shown on the right-hand panels starts after positioning the inlet. Table 4 lists the mean gas phase concentrations of each solvent for the spraying period and the highest concentrations reached during evaporation (blue sections), the maximum concentration reached by a spike and the concentration before the measurement was stopped.

**Table 4:** Mean solvent concentrations in ppmv with standard deviations averaged over the PTR-ToF-MS signal with 1 s time resolution (blue sections marked in Figures 3–5), the time in seconds when the maximum of drying was reached, and the end concentration of the measurements.

**Experiment**	**Task (Time interval)**	**Acetone m/z = 60**	**Ethanol m/z = 45**	**Butyl Acetate m/z = 117**	**Xylene m/z = 107**	**1M2PA m/z = 133**
Open door experiment	Spraying (100 s)	400 ± 272	189 ± 129	60 ± 48	70 ± 44	10 ± 7.4
	Maximum of drying (10 s)	85 ± 15	49 ± 7.2	121 ± 25	24 ± 3.7	12 ± 3.9
	(Start time of maximum)	(117 s)	(117 s)	(117 s)	(117 s)	(117 s)
	End of drying (120 s)	0.2 ± 0.2	0.8 ± 0.2	0 ± 4.3	0.1 ± 0.03	1.9 ± 2
Partially open-door (4 cm) experiment	Spraying (79 s)	369 ± 375	119 ± 118	20 ± 22	53 ± 60	5.8 ± 5.6
	Maximum of drying excluding spikes (60 s)	124 ± 15	48 ± 5.4	184 ± 54	49 ± 5.7	11 ± 7.8
	(Start time of maximum)	(123 s)	(206 s)	(620 s)	(266 s)	(698 s)
	Maximum of drying including spikes (7 s)	69 ± 5.5	172 ± 11	-	79 ± 4.6	-
	(Start time of the spike)	(350 s)	(350 s)	-	(350 s)	-
	End of drying (120 s)	0.7 ± 0.3	1.7 ± 0.4	3.3 ± 8	0.6 ± 0.1	2.3 ± 2.6
Closed door experiment	Spraying (71 s)	615 ± 554	178 ± 162	21 ± 15	74 ± 65	39 ± 36
	Maximum of drying excluding spikes (120 s)	170 ± 23	51 ± 4.4	132 ± 38	77 ± 9.8	75 ± 15
	(Start time of maximum)	(301 s)	(907 s)	(450 s)	(718 s)	(2,696 s)
	Maximum of drying including spikes (14 s)	233 ± 21	74 ± 5.3	-	127 ± 10	-
	(Start time of the spike)	(682 s)	(682 s)	-	(682 s)	-
	End of drying (120 s)	59 ± 10	17 ± 2.7	32 ± 46	30 ± 4.1	45 ± 17

### 3.1 Time-resolved concentrations measured during spray paint application

As spray painting was always performed in front of the container in the same manner, we are able to compare the three results to evaluate the reproducibility of the spraying process. For acetone, ethanol, and xylene, the left columns of Figures 3–**5** show strongly varying concentrations within one application and between the three applications with almost the same pattern of peaks for all three solvents. Butyl acetate and 1M2PA, on the other hand, exhibit a much weaker and noisy gas-phase signal during spraying. Assuming that the gas phase concentration during spraying is dominated by the evaporation of overspray droplets with only minor contributions from evaporation from the plate, the strong variations in gas-phase concentrations of acetone, ethanol, and xylene can be explained by the applied line-by-line pulsed spraying method together with air movements and wind, which blew the overspray away from the inlet in an irregular pattern. This pattern is much weaker or even absent for butyl acetate and 1M2PA, which shows that these solvents hardly evaporated during spraying and confirms that PTR-ToF-MS measured exclusively the gas phase with no droplets entering the inlet. The large variability in the measured concentrations of acetone, ethanol, and xylene explains the large standard deviation in Table 4 for these solvents during spraying. In comparison, the standard deviations of butyl acetate and 1M2PA are smaller due to the noisiness of their weak signal, which is owed to the lower sensitivity of PTR-ToF-MS to esters. Considering all this, the mean concentrations of the solvents during spraying show reasonable agreement with each other. Nevertheless, the differences in average exposure vary considerably. The concentration of acetone, ethanol, and xylene vary all by a factor of about 1.5, butyl acetate by a factor of 3, and 1M2PA even by 6.7, when we compare the three sprayings.

### 3.2 Drying dynamics and ventilation conditions

The drying process varied significantly depending on the ventilation conditions. For the open-door experiment (Figure 3), the gas phase concentrations show a near-exponential decay for all solvents and reach constant values within the measurement uncertainties after 1,000 s (around 16 min). Comparing the end concentrations with the average outdoor signal before the measurement started (acetone: 0.2 ± 0.2 ppmv; ethanol: 1.2 ± 0.4 ppmv; butyl acetate: 0.6 ± 6.3 ppmv; xylene: 0.04 ± 0.02 ppmv; 1M2PA: 1.9 ± 2 ppmv) shows that they correspond to background values. The rather high background signal and uncertainties can be explained by the dilution step that was applied to measure the high concentrations during spraying, because converting the values back to the real concentrations increased the noise level. Comparison of spraying and drying signal intensities shows that the main exposure to acetone, ethanol, and xylene occurred during spraying. For butyl acetate and 1M2PA, the maximum measured signals during the drying phase were above the average signal during spraying, as these substances build up only slowly during spraying.

For partially-open door during drying (4 cm) (Figure 4), acetone shows again a near-exponential decay in the gas phase concentration, while the concentrations of ethanol, butyl acetate, xylene, and 1M2PA first exhibit an increase followed by a near-exponential decay, which is clearly slower than the one for the open-door situation. Thus, the maximum concentration during drying was reached later and persisted longer. For butyl acetate and 1M2PA, it took around 10 min to reach the maximum concentration, which by then clearly topped the concentration reached during spraying (see Table 4, maximum of drying). Butyl acetate levels remained above the concentration reached during spraying for over 30 min. The maximum in gas-phase concentrations observed for butyl acetate, xylene, and 1M2PA can be explained by their relative increase in terms of mole fraction within the paint layer due to evaporation of the more volatile solvents acetone and ethanol, leading to an increase of partial vapor pressures. The gas phase concentrations of the solvents at the end of the measurement after about 11,300 s (about 188 min) are slightly higher than the values measured for the open-door experiment, maybe because of ongoing evaporation or slow diffusion out of the container.

When the door was closed during drying, all solvents showed first an increase before their concentrations started to decrease. Therefore, an increase in partial vapor pressure of some components at the expense of the others cannot fully explain this behavior. Rather, slow gas-phase diffusion seems to be relevant, having led to a time delay between evaporation from the plate and reaching the inlet of the instrument. Gas-phase diffusion limitations are confirmed by the spikes that appeared for all solvents simultaneously in the mass spectra. We ascribe these to eddy diffusion, causing direct motion of air from the plate to the instrument inlet. These air flows therefore reflect the higher solvent concentration in the vicinity of the plate surface compared with the lower average concentration close to the inlet.

After having reached the maximum, the solvent concentrations did not show an exponential decrease, but rather a linear or irregular one. Moreover, all solvents except butyl acetate were still decreasing in concentration at the end of the measurement time after about 10,400 s (173 min). As the air conditioning system was not connected to outdoors, air was just recirculated within the room thus stimulating eddy diffusion. The horizontal gray bars in Figure 5 show the estimated level of the solvents assuming an airtight room. Their width reflects the uncertainties in the composition of the paint as disclosed in the safety data sheet, and the estimated loss of paint to overspray during outdoor spraying, which we assumed to be 40–60 % for an airless spray (34). The concentrations of acetone and butyl acetate are well within this uncertainty range in accordance with a homogeneous distribution in the container, while concentrations of ethanol and xylene are just approaching the gray bar, and 1M2PA is even above it, pointing to continuing evaporation of these solvents from the plate after the measurement was stopped.

Another observation during the closed-door drying phase was a sudden decrease in ethanol intensity after about 5500 s (around 92 min), where also the occurrence of spikes ended. We had to perform the closed-door experiment twice because the first time, the spray can turned empty in the middle of spraying, requiring switching to a new one, which was not shaken before spraying. Nevertheless, we share these results in SM to show that in this experiment, all solvents showed a clear decrease in evaporation rate also after 5,500 s (around 92 min), evidencing that this feature does not seem to be accidental but might be due to an abrupt decrease of diffusion within the paint layer, potentially due to a discontinuity in the drying process, e.g., through film formation on top of the paint layer. Note that the slight increase in butyl acetate concentration after 5,500 s (around 92 min) might be an artifact because the concentration of this ester could only be evaluated after the 1M2PA concentration was subtracted from the butyl acetate parent peak, constituting a source of increased uncertainty and bias.

In a next step, we compared the measured solvent concentrations during spraying with predictions from the exposure models ECETOC TRA (v3), Stoffenmanager (v4), and ART (1.5). We took the models activity-based estimate exposures (480 min) at different percentile levels. To compare with our measurements, we selected daylong spraying (>4 h) for ECETOC TRA. The TRA exposure results represent the 75th percentile of the calculated exposure (7). For ART and Stoffenmanager, we selected 50th and 90th percentiles, the latter one being the recommended percentile under REACH for risk characterization (3). For comparison with the model predictions, we assumed spraying for a dayshift with the mean concentration measured during the 70–100 s actual spraying time. All input parameters for TREXMO are listed in Table 3 and the comparison between measurements and model predictions are shown in Table 5. Note that we converted the exposures given in mg/m^3^ by the models to ppmv for easier comparison with measurements.

**Table 5:** Comparison between measured mean solvent concentration levels in ppmv during spraying and exposures predicted by the models ECETOC TRA, Stoffenmanager, and ART.

**Experiments/substances**	**Acetone m/z = 60**	**Ethanol m/z = 45**	**Butyl Acetate m/z = 117**	**Xylene m/z = 107**	**1M2PA m/z = 133**
Spraying 1	400	189	60	70	10
Spraying 2	369	119	20	53	5.8
Spraying 3	615	172	21	74	39
ECETOC TRA (v3) 75 %-ile (Min, Max)	420, 700	140, 140	70, 210	70, 210	70, 70
Stoffenmanager (v4.0) 50 %-ile (Min, Max)	39, 56	7.4, 17	0.7, 2.5	0.7, 2.5	0.4, 1.0
Stoffenmanager (v4.0) 90 %-ile (Min, Max)	352, 509	67, 157	6.5, 22	6.7, 23	3.7, 8.7
ART (v1.5) 50 %-ile (Min, Max)	4,210^*^, 4,210^*^	1,274, 2,760	10, 27	16, 60	3.7, 4.8
ART (v1.5) 90 %-ile (Min, Max)	4,210^*^, 4,210^*^	5,307^*^, 5,307^*^	72, 183	106, 391	24, 31
Swiss MAK values^**^	500	500	100	100	50
Derived No-Effect Level (DNEL)^***^- short	1,019	1,008	126	67	51
Derived No-Effect Level (DNEL)^***^ - long	509	504	63	18	51

(Min, Max)-values refer to composition uncertainty (see Table 1).

^*^Maximum concentration output from ART (35).

^**^Limit values in the workplace: Current MAK and BAT values (suva.ch) values of 2023.

^***^0013 (Regulation (EC) No. 1907/2006, Annex 2).

ECETOC TRA, which should, as a Tier 1 model, provide a conservative estimate of exposure, does not fully reach this goal for all solvents as also reported by Savic et al. (4). Specifically, the predicted exposure to ethanol is slightly underestimated for two sprayings. Nevertheless, ECETOC TRA predicts all solvents in the right concentration range. Note that for this model, the paint composition cannot be entered exactly but just in terms of >25 %, 5–25 %, 1–5 %, and < 1 %.

Stoffenmanager, the Tier 1.5 model, shows a difference of around one order of magnitude between exposure estimates for the 50th percentile compared with the 90th, with the predictions at the 50th percentile being clearly too low when compared to the measured values. For the 90th percentile, Stoffenmanager predicts all solvents in the right concentration range, albeit the less volatile ones (butyl acetate, xylene, 1M2PA) too low. One obstacle for accurate predictions is the wide concentration range given in the safety data sheet for the paint composition, leading to differences in prediction of more than a factor of 3 for butyl acetate and xylene (Table 4, [Min, Max] values). Thus, the advantage of entering the composition exactly is counterbalanced by the imprecise composition disclosed in the safety data sheets. Note that we used in this study Stoffenmanager (v4), which version incorporated in TREXMO. We repeated these calculations with the latest version of Stoffenmanager available online (v8, gestis.stoffenmanager.com) and found that the output is the same.

Finally, the Tier 2 model ART provides the upper ceiling values for the more volatile solvents acetone and ethanol for both the 50th and 90th percentiles, while for the less volatile substances butyl acetate, xylene, and 1M2PA the predictions are low compared with the measured values for the 50th and rather higher than measurements for the 90th percentile. ART is conservative for all solvents except for 1M2PA, for which the third spraying exceeds the upper estimate.

All solvents remained below the OEL during spraying except for acetone, which exceeded the short-term OEL during Spraying 3. Xylene exceeded the short-term DNEL value during two sprayings and the long-term DNEL value during all sprayings. Yet, the measured exposures would only be realized when spraying lasted for 8 h. Yet, the sum indices (*SI*) of the combined exposure to all solvents, clearly exceeded the allowable concentration (*SI* < 1) for all three sprayings, reaching values of 2.7 (spraying 1), 1.8 (spraying 2), and even 3.3 (spraying 3).

## 4 Discussion

This study presents a novel approach to assess workplace exposure during spray applications, using PTR-ToF-MS to monitor solvent concentrations in real time (22, 23). Our results show that this technique provides a comprehensive picture of exposure dynamics, covering both the spraying and the drying. With this approach, two goals can be reached, (i) giving process level-insights in spray applications that cannot be reached if only average concentrations during an arbitrary time period are measured; and (ii) providing reliable datasets for exposure model validation and development.

### 4.1 Relevance of spraying and drying for exposure to vapors

This study shows that online monitoring in spray applications can provide process-level insights that cannot be obtained by offline analysis. Owing to the small air volume of the container, we performed the spraying outdoors and then moved the freshly sprayed plate and the PTR-ToF-MS inlet indoors to monitor the drying process. Like this, the drying was not influenced by the dispersing overspray cloud. This procedure allowed us on one hand to obtain the spraying in replicate for comparison between each other, and, on the other hand, to investigate the role of ventilation by varying strengths of fresh air supply.

Online monitoring of the spraying process revealed that the gas-phase exposure during spraying strongly depends on the vapor pressure of the substances with the more volatile ones strongly dominating the total exposure. Conversely, exposure during drying is strongly influenced by the ventilation conditions. If drying takes place in a small room with no or limited outdoor air-exchange, gas-phase concentrations of the less volatile solvents build up and, as drying proceeds, start to dominate the total exposure to solvent vapors. These findings are supported by a recent study from Ding et al. (32), who measured real-time worker's exposure during Covid-19 disinfection activities (spraying, wiping, drying off). In 30, the PTR-ToF-MS inlet was connected within the breathing zone (about 10 cm from the nose) on the researcher's working suit while he moved within the room to disinfect different indoor spaces in a small bathroom by spraying a thymol- and plant-based disinfectant for a total of 10 min, followed by wiping each location dry for additional 10 min and an additional 60 min measurement period to register the decay. Like our results, this study found highly varying concentrations of the more volatile terpene components during spraying, while the concentration of the less volatile thymol peaked at the end of the wiping period. Both substances then show near-exponential decay. Like in our study with five solvents, gas-phase exposure to the less volatile substances became more relevant during drying than it was during spraying. Such detailed and time-resolved measurements offer a database to improve the understanding of spraying applications on a process level.

### 4.2 Implications for exposure models

Comparison of spraying measurements with predictions from exposure models showed that ECETOC TRA (v3) predicts concentrations in the measured range for all solvents, albeit not conservative for all three sprayings and all solvents (acetone, ethanol, xylene, butyl acetate, and 1M2PA). In previous studies, ECETOC TRA has been criticized for not being sufficiently conservative for industrial spraying applications (1). Landberg et al. (36) reported the underprediction of exposure by ECETOC TRA (v3) for chassis spray painting. Interestingly, the substance that was underpredicted was xylene, which was predicted in the right concentration range in our spray-painting application. In a broader study covering occupational exposure situations including spraying, Spinazzè et al. (37) found the performance of ECETOC TRA (v3.1) to be not acceptable in terms of accuracy for exposures to organic solvents and pesticides, as it led to too high as well as too low exposure estimates. However, we found neither large over- nor large underestimates in our spray application for the substances analyzed.

When Stoffenmanager, the Tier 1.5 model, was evaluated at the 90^th^ percentile, it predicted the more volatile solvents in the measured concentration range, yet it rather underestimated exposure especially for the less volatile solvents butyl acetate, xylene, and 1M2PA. For the 50th percentile, predictions were clearly too low. Previously, Landberg et al. (36) also tested Stoffenmanager for a chassis spray painting application and found xylene to be underpredicted, yet in the right concentration range when the 90th percentile was used. For the 50th percentile, the concentration was clearly underpredicted (19), which is in agreement with our findings.

ART, the Tier 2 model, overpredicted the more volatile solvents acetone and ethanol considerably while the less volatile ones were in the right concentration range. Overall, ART was the most conservative model for all solvents but 1M2PA, for which ECETOC TRA was more conservative. This outcome agrees with Landberg et al. (36), who concluded that ART was the most conservative model when compared with ECETOC TRA and Stoffenmanager. This is in contrast to the expectation that ART should be the least generic and most accurate model, as was also found by Savic et al. (4). Instead, ECETOC TRA, which is supposed to be conservative, was the least.

We did not compare the drying phase with model predictions because drying is not covered by ECETOC TRA and ART. Only Stoffenmanager offers the option to model drying, but only in conjunction with spraying and under the same ventilation conditions. Thus, our experimental setting of outdoor spraying and indoor drying is not covered. However, this study shows that a comprehensive description of spraying, including drying, is urgently needed in exposure models to capture high exposures to less volatile solvents during the drying phase, especially when ventilation conditions are not ideal.

## 5 Conclusion

In this study we used a PTR-ToF-MS system to monitor the gas-phase concentration during spraying and drying of a spray can paint. We established a dataset that consists of the time-resolved gas-phase concentration of acetone, ethanol, xylene, butyl acetate, and 1M2PA during spraying performed three times outdoors, always in the same manner, and the evolution of the concentration of the same solvents while the paint was drying indoors in a container. For the drying phase, we varied the ventilation conditions each time: spraying 1 was performed with open door, spraying 2 with partially open door, and spraying 3 with closed door.

Owing to the high time resolution of PTR-ToF-MS, the measurements revealed strongly fluctuating overspray concentrations during spraying, leading to an average exposure that varied by a factor of 1.5 for acetone, ethanol, and xylene, and even by a factor of more than 6 for 1M2PA when comparing the three sprayings. For this reason, measuring and modeling must first be compared neutrally: measurements may be very accurate at a particular location and time, yet, they might not be very representative. Conversely, modeling at the given place and time may not by perfect, but may be more representative.

For the drying phase, we observed a strong influence of the ventilation conditions: for acetone and ethanol, the average gas phase concentration during spraying was higher than the maximum concentration during drying for all ventilation conditions; for xylene, butyl acetate, and 1M2PA, the maximum concentration during drying was equal to the average concentration during spraying with the door partially open, and for butyl acetate and 1M2PA, the concentration during drying clearly exceeded the exposure during spraying with the door closed. This highlights the relevance of drying for estimating total exposure to spray paints when ventilation conditions are not ideal. Hence, we recommend that drying should be integrated into the model predictions.

Comparison of the spraying phase with exposure model predictions showed that ECETOC TRA (v3) and Stoffenmanager (v4) predicted exposure in the measured concentration range, albeit not conservative for all solvents and all sprayings. On the contrary, ART (v1.5) strongly overestimated the exposure for the more volatile solvents acetone and ethanol and slightly underestimated exposure to 1M2PA for one spraying. Again, the large variability of overspray vapor concentrations due to random air flows during the outdoor spraying highlights the difficulty in acquiring a representative database as input for exposure models when measurement conditions are very random (e.g. due to wind or variable air circulation).

As an important result of this work, it became clear that more attention should be paid to the drying phase, especially when the less volatile solvents are the more hazardous ones and when ventilation conditions are not ideal. It should be noted that evaporation of less volatile solvents during product drying is not limited to spraying applications but also occurs during wiping, brushing, rolling, or mopping. Some of these activities may well be performed in small spaces with limited ventilation. Therefore, the inclusion of the drying phase in exposure model predictions is strongly warranted. Also here, online-monitoring techniques such as PTR-ToF-MS should be considered as the methods of choice for model development and improvement.

## Data availability statement

The raw data supporting the conclusions of this article will be made available by the authors, without undue reservation.

## Ethics statement

Written informed consent was obtained from the individual(s) for the publication of any potentially identifiable images or data included in this article.

## Author contributions

SS: Formal analysis, Investigation, Methodology, Resources, Software, Validation, Visualization, Writing—original draft, Writing—review & editing. DB: Formal analysis, Investigation, Methodology, Resources, Software, Validation, Writing—review & editing. BG: Conceptualization, Funding acquisition, Writing—review & editing. KS: Conceptualization, Funding acquisition, Writing—review & editing. TP: Funding acquisition, Supervision, Writing—review & editing. CM: Conceptualization, Funding acquisition, Project administration, Supervision, Validation, Writing—original draft, Writing—review & editing.
