# Supplementary material for: Exposure assessment during paint spraying and drying using PTR-ToF-MS
Source: Front Public Health. 2024 Jan 12;11:1327187. doi: 10.3389/fpubh.2023.1327187 (PMC10811262; doi:10.3389/fpubh.2023.1327187)
Supplement: Supplementary file 1 [file Data_Sheet_1.docx]

**Supplementary material**

**Exposure assessment during paint spraying and drying using PTR-ToF-MS**

| Supplementary Table S1. Volumes of air used for dilution experiments, for measurement of standards and spraying experiments. | | | | | |
| --- | --- | --- | --- | --- | --- |
| Substance | **Input (a)**  **[L/min]** | **Zero air 1 (b) [L/min]** | **Exhaust 1 (c) [L/min]** | **Zero air 2 (d) [L/min]** | **PTR-ToF-MS inlet (e) [L/min]** |
| Acetone | 0.03 | 9 | 8.95 | 8 | 0.4 |
| Ethanol | 0.03 | 9 | 8.95 | 8 | 0.4 |
| Xylene | 0.03 | 9 | 8.95 | 8 | 0.4 |
| Butyl acetate | 0.03 | 9 | 8.95 | 2 | 0.4 |
| 1-Mehoxy-2-propyl acetate (1M2PA) | 0.03 | 9 | 5 | 4 | 0.4 |
| Spray can paint | 0.05 | 8 | 8.35 | - | 0.4 |

**Setup for real-time VOC measurements**

For our measurement setup shown in Fig. 2 of the main text, we used PFA tubing (6 mm in diameter) for the lines and wider steel tubing for the mixing regions (these regions are designed to facilitate the thorough mixing of gases, allowing for homogeneity and consistent composition of the airflow). We incorporated mass flow controllers to regulate the volume of zero air entering and exiting the PFA tubing that leads to the instrument.

**Description of the container**

The container held several measuring devices, the inside walls are made of PVC and the floor out of wood coated with a resin. During the experiments there were three metal racks in it, two racks were connected to a wall and covered it completely. On the third rack, there were three measuring devices used for another study. Moreover, there were different tools and accessories for the measurement equipment. As already mentioned in the manuscript, the container has three air conditioning units. Windows were closed and there was no interaction with sunlight.

We cannot give an air exchange rate for the open and semi-open-door experiments as the flow through the door could not be quantified. For the closed-door scenario, there was no exchange with outdoor air, because the air conditioner mounted in the container is not connected to the outdoor and just leads to a mixing of the indoor air. Instruments in the container had a specified output to the outdoor of 2 L/min, which in terms of Air Change per Hour (ACH) is negligible.

**Chamber measurements**

The experiments were conducted within the indoor reaction chamber situated at the Paul Scherrer Institute (PSI). This PSI reaction chamber, a transparent Teflon® bag with a volume of 9 m³, is suspended within a temperature-controlled housing. Known volumes of the components were injected into a heated port where 100 L/min of clean air passed over the sample. Concentrations were calculated given the volume of the chamber (9 m^3^) and using the component density and volume to determine their mixing ratios.

Species that were injected with their respective quantities are presented in Table S2.

| Table S2. Concentrations of the injected standards and the calibration factors that were calculated from them. | | | |
| --- | --- | --- | --- |
| Substance | **Injected in chamber [ppbv]** | **Measured [ppbv]** | **Calibration factor [CF]** |
| Acetone | 101.8 | 72 | 1.5 |
| Ethanol | 298.3 | 31.5 | 9.5 |
| Butyl acetate | 94.3 | 0.06 | 1594 |
| Xylene | 100.9 | 9.6 | 10.5 |
| 1-Mehoxy-2-propyl acetate (1M2PA) | 90.4 | 0.06 | 1481 |

**Transmission efficiency curve**

In our study, we carefully corrected signal intensity by accounting for transmission efficiency. We have performed calibrations with a commercially available calibration gas bottle with specified concentrations of gases in it. This correction is crucial, considering the impact of mass-to-charge ratio (m/z) on ion counts transmitted from the drift tube to the detector. The correction process is visually depicted in a figure S1, underscoring its importance in ensuring accurate and reliable intensity recordings in our mass spectrometry analysis. This approach enhances the precision and validity of our research findings.


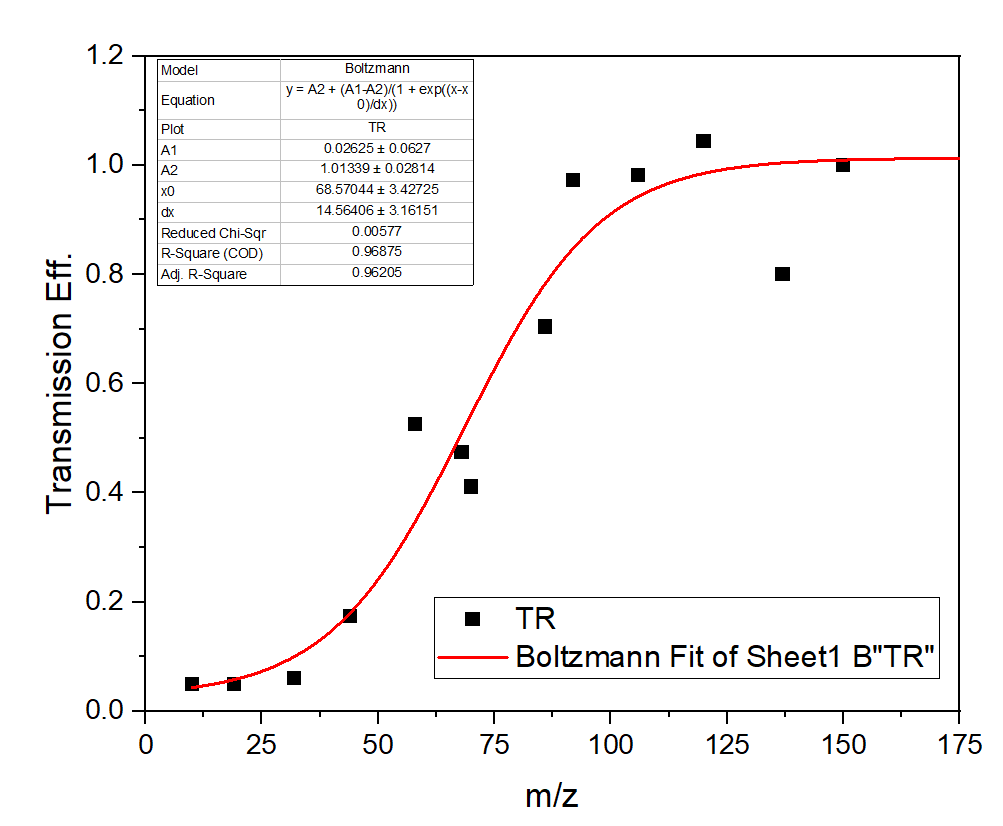


Figure S1. Transmission efficiency curve obtained by using a Boltzmann Fit of measured dependency of ion counts transmitted from the drift tube to the detector as a function of the mass-to-charge ratio (m/z).

**Preparation for spraying of the paint**

In strict adherence to the manufacturer's stipulations, the protocol necessitated a thorough mixing of the contents within the spray can for a duration of 120 seconds prior to start of the spray application process. The ensuing procedure involved initiating the spray from an approximate distance of 20 cm from the designated metal plate, characterized by a methodical horizontal motion, progressing row by row. Subsequently, there was a controlled descent in the trajectory for each subsequent horizontal pass, a technique employed until the complete surface achieved a uniformly painted state.

During the experimental process delineated in Figure S2, an interim necessitated a transition of the spray can due to the initial spray can nearing depletion. This transition occurred at the midway juncture of the painting process, wherein a replacement spray can was introduced. Notably, it warrants mention that the second spray can, having been in a state of inactivity during the utilization of the initial spray can, was not subjected to the same degree of agitation prior to application. This temporal inactivity potentially underlies the observed disparity in concentration levels throughout the phases of both spraying and drying in the course of this particular experiment.

From the first spray can, we have applied 39 g of product and from the second we have applied 43 g of product.

This deviation in procedural adherence bears significance in explains the variance in concentration levels, underscoring the pivotal role of rigorous protocol adherence in ensuring consistency and accuracy in experimental outcomes.


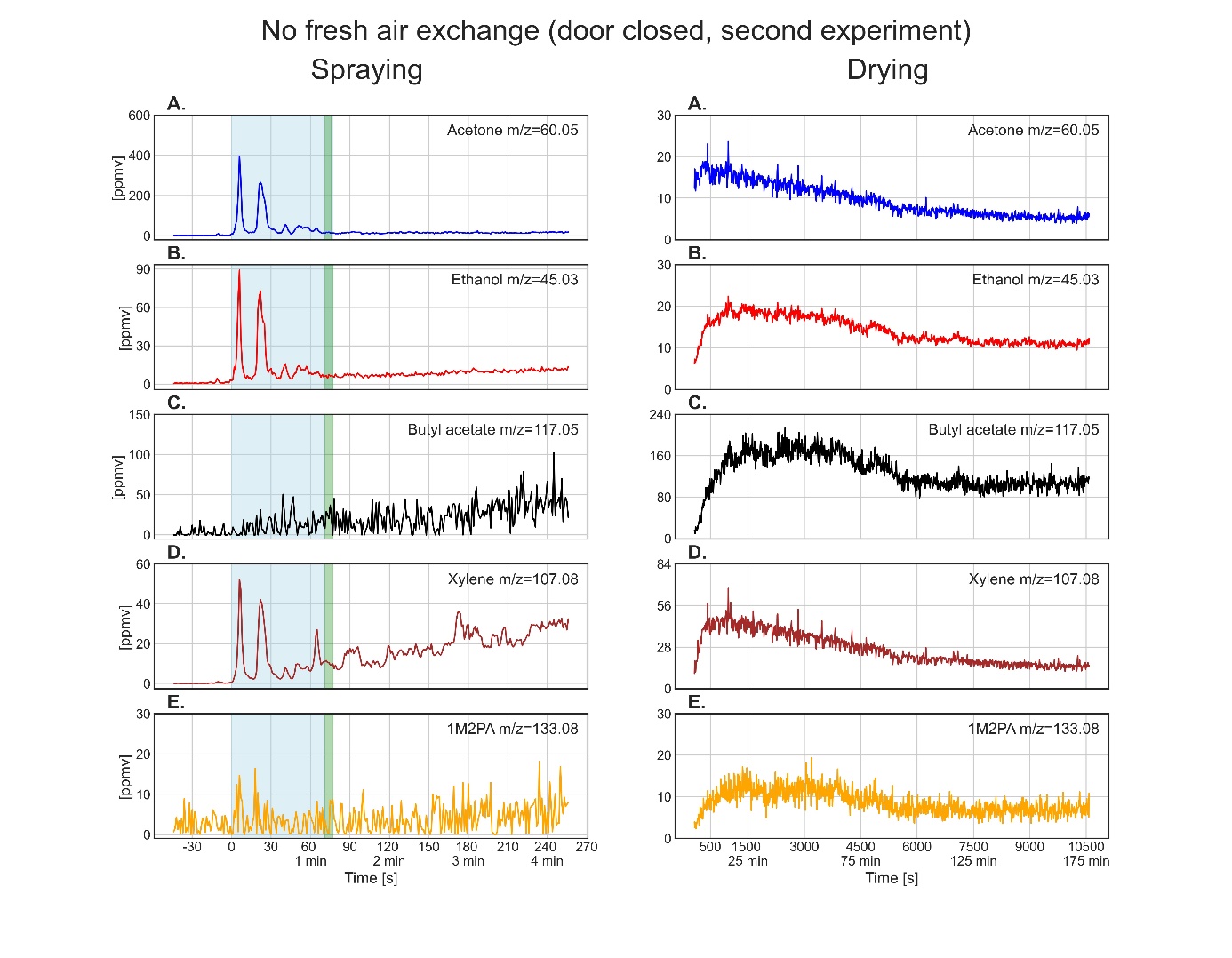
Supplementary Figure S2. Time series of monitored solvents in order of their vapor pressures (from high to low) for the spray paint application with closed door during drying. In the left column, spraying is shown. The start of drying, in the right column, was set to 77s. The light blue shaded regions in the left column mark the effective spraying period. The green shaded area represents the transfer of the metal plate and PTR-ToF-MS inlet indoors.
